# Authors' Response to the Letter by Sun et al.

**DOI:** 10.1111/hex.70564

**Published:** 2026-05-11

**Authors:** Hannah Rutherford, Marita Dale, Sally Wootton, Rashmi Pithavadian, Sarah Dennis, Sarah Brown, Jennifer A. Alison, Andrew S. L. Chan, Ian A. Yang, Zoe McKeough

**Affiliations:** ^1^ Sydney School of Health Sciences, Faculty of Medicine and Health University of Sydney Sydney New South Wales Australia; ^2^ Directorate of Strategy, Innovation and Digital Health, South Eastern Sydney Local Health District Sydney New South Wales Australia; ^3^ Kolling Institute, Faculty of medicine and Health University of Sydney New South Wales; ^4^ Chronic Disease Community Rehabilitation Service, Northern Sydney Local Health District, North Ryde Sydney New South Wales Australia; ^5^ School of Health Sciences Western Sydney University Campbelltown New South Wales Australia; ^6^ South West Sydney Allied Health Research Collaboration, South West Sydney Local Health District Liverpool New South Wales Australia; ^7^ Ingham Institute for Applied Medical Research Liverpool New South Wales Australia; ^8^ Department of Physiotherapy Royal North Shore Hospital, St Leonards Sydney New South Wales Australia; ^9^ Allied Health, Sydney Local Health District Sydney New South Wales Australia; ^10^ Department of Respiratory and Sleep Medicine Royal North Shore Hospital, St Leonards Sydney New South Wales Australia; ^11^ Northern Clinical School, Faculty of Medicine and Health The University of Sydney Sydney New South Wales Australia; ^12^ The Prince Charles Hospital and Faculty of Medicine The University of Queensland Brisbane Australia

## Response

We sincerely appreciate the thoughtful comments and interest expressed by Sun et al. regarding our qualitative study. Their engagement highlights the importance of exploring patient experiences in pulmonary rehabilitation (PR) research. Our qualitative study complements findings from our randomised controlled equivalence trial (RCT) [1] that included 90 participants. Our RCT demonstrated that a mHealth PR programme resulted in equivalent improvements in exercise capacity and superior improvements in health status when compared with centre‐based PR (CB‐PR) in people with chronic obstructive pulmonary disease (COPD). The aim of our qualitative study was to explore the participant experience of the mHealth programme and how it compares and differs from CB‐PR. Due to the often low uptake and completion rates of CB‐PR [2, 3, 4], we suggest PR providers consider the participant's experience of different service models alongside the evidence for effectiveness when prescribing PR.

We clearly acknowledge in our manuscript that the transferability of our findings may be limited to individuals who share characteristics outlined in our RCT inclusion criteria. We specifically state that the results may not be generalisable to populations from different cultural backgrounds or to those with limited digital literacy. We also note in our limitations that we did not assess nutritional intake and baseline involvement in exercise. However, we note the majority of the cohort were ex‐smokers with moderate to severe COPD and multiple comorbidities, reflecting the typical patient population referred to PR programmes in Australia. Additionally, the sample of participants invited to interview was representative of the cohort from the RCT, including by age (average of 75 [SD 7] years) and with greater than 85% of the cohort retired. The sample size was appropriate for a qualitative study design, which aimed to complete an in‐depth, inductive exploration of the participant experience and was determined based on data saturation. Our focus was on achieving thematic depth across a range of participant contexts, rather than generalisability. We note in our manuscript the strategies used to ensure the qualitative trustworthiness of our results.

In regard to a long‐term follow‐up plan, an 8‐week exercise programme was examined as this is in line with national and international guidelines [3, 5] and is the commonly prescribed length of programme in Australia. Long‐term sustainability of results was beyond the scope of these two studies, and we agree that further research is required to evaluate the maintenance of outcomes of mHealth PR programmes.

## Author Contributions

All authors read and approved the letter of response.

## Conflicts of Interest

The authors declare no conflicts of interest.

## Data Availability

The data that support the findings of this study are available from the corresponding author upon reasonable request.
